# Women’s Mental Health as a Factor Associated with Exclusive Breastfeeding and Breastfeeding Duration: Data from a Longitudinal Study in Greece

**DOI:** 10.3390/children8020150

**Published:** 2021-02-17

**Authors:** Maria Dagla, Irina Mrvoljak-Theodoropoulou, Despoina Karagianni, Calliope Dagla, Dimitra Sotiropoulou, Eleni Kontiza, Aikaterini-Taxiarchoula Kavakou, Evangelia Antoniou

**Affiliations:** 1 Day Center for the Care of the Mental Health of Women (Perinatal Mental Health Disorders), Non-Profit Organization “FAINARETI”, 17121 Athens, Greece; imrvoljak@hotmail.com (I.M.-T.); dekarag@gmail.com (D.K.); aebmc18007@uniwa.gr (C.D.); dswtir@gmail.com (D.S.); helenakontiza@gmail.com (E.K.); kavakoukat@gmail.com (A.-T.K.); lilanton@uniwa.gr (E.A.); 2 Department of Midwifery, University of West Attica, 12243 Athens, Greece

**Keywords:** mental health, breastfeeding, duration, exclusivity, depression, EPDS, PHQ-9

## Abstract

Background: This study investigated the relationship between exclusive breastfeeding and breastfeeding duration, and maternal psychological well-being in the perinatal period. Methods: A longitudinal study involving a retrospective follow-up of a group of 1080 women from pregnancy to the 1st year postpartum, who gave birth during the 5-year period between January 2014 and January 2019 in Athens, Greece, was designed. Women’s history and two psychometric tools—the Edinburg Postpartum Depression Scale (EPDS) and the Patient Health Questionnaire-9 (PHQ-9) administered at 5-time points—were used for data collection. Logistic regression analysis and a series of multiple analysis of variance (MANOVA) tests were performed. Results: The chance for exclusive breastfeeding (giving only breast milk) appeared to decrease (a) with an increase of the scores for psychometric tools antenatally (PHQ-9, *p* = 0.030) or at the 6th week postpartum (EPDS, *p* < 0.001 and PHQ-9, *p* < 0.001), (b) with an increase in the number of psychotherapeutic sessions needed antenatally (*p* = 0.030), and (c) when the initiation of psychotherapy was necessary postpartum (*p* = 0.002). Additionally, a shorter duration of any breastfeeding (with or without formula or other types of food/drink) seems to be associated with (a) the occurrence of pathological mental health symptoms (*p* = 0.029), (b) increased PHQ-9 scores antenatally (*p* = 0.018), (c) increased EPDS scores at the 6th week (*p* = 0.004) and the 12th month postpartum (*p* = 0.031), (d) the initiation of psychotherapy postpartum (*p* = 0.040), and e) the need for more than 13 psychotherapeutic sessions (*p* = 0.020). Conclusions: This study demonstrates a negative relationship between exclusive breastfeeding and breastfeeding duration, and poor maternal mental health in the perinatal period.

## 1. Introduction

Breastfeeding and a woman′s mental health in the perinatal period are two critical public health issues that significantly affect the quality and quantity of human life. Breastfeeding is the “ideal” way of feeding neonates, infants, and young children. It is a biological process with a protective effect on women and seems to be related to good physical and emotional health for the mother during the puerperium, the lactation period, and all her future life [1]. It has also been linked to a reduced risk of type 2 diabetes mellitus and breast and ovarian cancer [2], and to a weakened stress response, reduced anxiety, and reduced negative mood symptoms [3,4]. The World Health Organization (WHO) and many other authorities recommend that breastfeeding starts within the first hour of life, be exclusive for 6 months, and continues for up to two years of age or beyond [5]. Despite the proven benefits of breastfeeding and the international recommendations, breastfeeding rates in most countries (especially exclusive breastfeeding rates) remain low [6,7]. Among the factors that have been blamed for this situation are factors related to a woman’s mental health before and after birth [4]. 

The perinatal period (from pregnancy to 1st year postpartum) is a crucial period for the development of mental health disorders in women, such as anxiety disorders, mood disorders, psychoses, etc. Epidemiological data have shown that perinatal major depressive disorder affects at least 20% of women [8,9] and perinatal anxiety affects 10% [8], while approximately 10–15% of women report moderate depressive symptoms during pregnancy and/or the postpartum period [10]. These symptoms may not have been recognized or diagnosed by the woman herself, her family, or a specialist. Perinatal mental health disorders are among the most common morbidities of pregnancy and the postnatal period and seem to have a negative effect on perinatal care and birth outcomes [11,12,13] and on the cognitive, social, and emotional development of the child [14,15,16,17]. 

The international literature has associated breastfeeding with the new mother’s anxiety, mood, and emotional state. We generally know that, through breastfeeding, a woman′s body follows a “natural order” towards hormonal balance [18]. The breastfeeding process has anxiolytic and anti-depressive effects due to the release of oxytocin [19,20], which plays a role in the reduction of stress and anxiety [21], and, at the same time, due to a decrease of maternal adeno-corticotropic hormone (ACTH) and cortisol release levels [22], which supports the finding that lactation attenuates neuro-endocrine responses to stress [23]. The “protective” effect of breastfeeding on maternal mental health, as shown by the attenuation of stress responses [24,25], the reduced risk of developing depression, or mitigation of the severity of symptoms, has been supported through research [20,26,27]. Women who continued to breastfeed up to 4 months after giving birth had lower depression scores in the first month than those who stopped breastfeeding [28]. Women who do not start or continue breastfeeding are at risk of postpartum depression [4,20]. In addition, the early cessation and a shorter duration of breastfeeding seem to have a negative effect on a woman′s mental health, as they have been linked to the mother′s feelings of guilt, shame, and concern for the neonate’s health [29,30]. Breastfeeding also acts as a protective factor for a woman′s mental health and psychological well-being through its link to the regulation of sleep–wake patterns [31,32], improvement of the mother’s self-efficacy [33], mother–infant interaction [34], and other processes that affect the mother’s psychology. 

There are studies that refer to a two-way relationship between breastfeeding and the occurrence of depression [35,36], and others that consider this relationship to be controversial [37,38]. According to systematic reviews and other international research, women who had high levels of anxiety and/or depression before or during pregnancy or after giving birth (increased scores for psychometric tools that detect these health disorders) are more likely than others to have difficulty with breastfeeding [18,37,38,39,40,41]. These women will not start breastfeeding at all [37,42], will stop prematurely and will therefore breastfeed for a shorter period [18,28,37,40,41,43,44], or will exclusively breastfeed to a lesser extent and for a shorter duration than women who did not show such symptoms [28,44,45,46,47]. Therefore, the development of perinatal symptoms of anxiety and depression appears to be a risk factor for the non-initiation of breastfeeding, its discontinuation postpartum, and its early cessation, as well as the non-exclusivity of breastfeeding. Conversely, the early cessation of breastfeeding has been shown to have a negative effect on a woman’s psychology, as it has been associated with increased anxiety and depression symptoms within 6 months after delivery [26] and appears to be an aggravating risk factor, especially for women who are mentally burdened with pathological symptoms [26]. In general, low breastfeeding rates, a shorter duration of exclusive breastfeeding, and the cessation of breastfeeding before the completion of 6 months are associated with increased anxiety and/or depression and contribute to the mother’s disappointment [37,48,49,50].

On the other hand, there is literature that does not link breastfeeding to the presence of pathological symptoms of a mental health disorder during the perinatal period. The results of many prospective studies do not associate breastfeeding with depression [51,52] or depression with the discontinuation of exclusive breastfeeding [53]. Another study also argues that there is no two-way relationship between breastfeeding and depression [54]. The results of all studies examining the possible link between breastfeeding and the occurrence of a mental health disorder and vice versa show small to large differences in research methodologies, tools that detect mental health disorders, and definitions related to breastfeeding (exclusive or not). These differences, in combination with the variety in the periods investigated (e.g., some studies only investigate the 1st month postpartum, whilst others investigated up to the 4th or 6th month, and a few until the 12th month postpartum), as well as the variety of socio-cultural contexts of the samples, seem to contribute to the diversification of results [44].

As can be seen from the above, the association between breastfeeding and depression has not yet been precisely explained, the direction of this association is still unclear [55], and there are conflicting views that require further exploration. In addition, we do not know whether early breastfeeding cessation is predictive of the occurrence of a mental health disorder such as anxiety and/or depression postpartum or whether this happens with all women or only those who are mentally burdened. Additionally, the relationship between the process and duration of breastfeeding and the symptoms of anxiety, or the diagnosis of an anxiety disorder, has not been sufficiently investigated. The examination of the relationship between breastfeeding and perinatal mental health disorders other than depression is considered imperative for public health, given the serious adverse effects of both the non-initiation or early cessation of breastfeeding (exclusive or not) and the occurrence of a mental health disorder in the perinatal period. 

The objective of this study is to investigate the relationship between exclusive breastfeeding and breastfeeding duration, and maternal psychological well-being and the presence of a perinatal mental health disorder, from pregnancy until the 1st year postpartum. More specifically, this study highlights whether exclusive breastfeeding and the breastfeeding duration are associated with specific maternal psycho-emotional factors in the perinatal period. It is one of a few cases where a study on breastfeeding (a) investigates the role of such a large number of psycho-emotional and psychiatric factors in exclusive breastfeeding and the breastfeeding duration; (b) investigates the effect of these factors for such a long and continuous period as opposed to only during the early postpartum period, as is usually the case; and (c) takes into account the psycho-emotional state of the woman in the past, even before the perinatal period.

## 2. Materials and Methods

### 2.1. Study Population

A longitudinal study involving a retrospective follow-up of a group of women from pregnancy to the 1st year postpartum was designed. This study is based on data from 1080 women who gave birth during the 5-year period between January 2014 and January 2019 in Athens, Greece. All the women had participated in an innovative psychosocial health intervention implemented at the Day Center for the Care of the Mental Health of Women (Perinatal Mental Health Disorders), representing the only Day Center operated in the country by the Non-Profit Organization FAINARETI. This Day Center is a primary community facility, supervised and funded by the Mental Health Department of the Greek Ministry of Health. The admission criteria set in this study required that (a) the woman had completed the Day Center’s psychosocial health intervention, (b) there were sufficient data for her (e.g., a complete history), and (c) she spoke the Greek language. All women who participated in the study had given their informed oral and written consent for the analysis of their data for research purposes and had been informed of their right to withdraw their consent at any point. This research study was approved by the Research Ethics Committee of the Non-Profit Organization FAINARETI (Ref. Number 77/03.07.19). 

### 2.2. Data Collection 

All women and their partners that attended the Day Center’s psychosocial health intervention were offered the following services during pregnancy and up until the 1st year postpartum: (a) A Midwifery-led Antenatal Educational Program called “Preparation for Labor and Parenthood” (8–12 three-hour group sessions or 4–5 two-hour individual sessions); (b) an Antenatal Physical Activity Program by a specialized physical education teacher (only to women who requested it); (c) midwifery-led counseling and support; (d) antenatal and postpartum screening for pathological mental health symptoms (at regular intervals); (e) counseling and support by mental health professionals (psychological assessment, monitoring and psychotherapy, psychiatric assessment, and treatment); (f) a training and support program for new parents at the 3rd, 6th, and 12th month postpartum; (g) phone support and counseling by mental health professionals and midwives; and (h) midwifery-led counseling or counseling by a mental health professional for families. The data analyzed in this study were obtained from (a) the medical, social, and psycho-emotional history of the woman; (b) the peripartum and postpartum health and well-being history; (c) a questionnaire on infant feeding and behavior; and d) the scores of the psychometric screening tools employed for the detection and evaluation of symptoms of perinatal mental health disorders. 

The participants’ demographic data, as well as their medical, social, and psycho-emotional history, were recorded by a midwife before the start of the psychosocial health intervention (approximately between the 18th and 22th gestation week) and after their oral and written consent had been obtained. Demographic data included the mother’s age, educational level, marital status, financial level, and religious beliefs. Information regarding the mother’s medical history included the presence of illness (chronic or not), surgeries, systematic or long-term use of drugs, conception through in vitro fertilization methods, family history of severe illness, etc. The social and psycho-emotional history included information on lifestyle, social support or alienation, job satisfaction, weight and sleep problems, the relationship with the partner, and the existence of traumatic life events. The same section examined the woman’s feelings and mood during the current pregnancy. Possible pathological symptoms, mood problems, anxiety, suicidal ideation, physical or psychological violence, psychotherapeutic sessions, and taking medication were also documented. This section provided information on possible mental health illness and risk factors in a woman’s family history. Through the medical, social, and psycho-emotional history, the necessary information was collected, in order to identify either current pathological mental health symptoms or risk factors that burdened the woman’s life, so as to refer her to a mental health specialist, if necessary. 

In addition, a peripartum and postpartum health history was taken at the 6th week postpartum. The main information obtained through this history included the manner of the initiation of labor, its type and duration, and the mother’s satisfaction with it, as well as factors related to the newborn, such as the gender, weight, possible admission to the neonatal intensive care unit, and type of feeding (exclusive or any breastfeeding, or feeding with formula) from the 1st day after birth (in the hospital) until the 6th week postpartum. This peripartum and postpartum history contained the first recorded information related to breastfeeding. It also included details on the new mother’s feelings and mood, possible pathological mental symptoms, sleep and/or fatigue problems, satisfaction with motherhood, relationship with their partner, and support from family.

At the end of the 1st year postpartum, information was received, retrospectively (via e-mail or telephone), regarding the infant’s feeding and behavior during the period from the end of the 6th week to the end of the 1st year postpartum. This was the second recording of breastfeeding data, i.e., the total duration of exclusive and any breastfeeding (in months), the point in time when solid foods were introduced, the possible difficulties with breastfeeding, infant sleep difficulties, etc. Mothers who continued to breastfeed beyond the 1st completed month postpartum were contacted (via e-mail or telephone) at 24 months postpartum, in order to determine for how long they continued to breastfeed after the completion of the 1st year (third recording of breastfeeding data). Throughout the psychosocial health intervention, repeated screening for pathological mental health symptoms and assessment of the well-being of the woman were performed at the following five time points: (a) Before the start of the midwifery-led antenatal educational program (approximately 24th–28th gestation week); (b) at the end of the midwifery-led antenatal educational program (approximately 34th–38th gestation week); (c) at the 6th week postpartum; (d) at the 6th month postpartum; and (e) at the 12th month postpartum. 

### 2.3. Screening Tools

The psychometric screening tools that were used will now be introduced. 

Τhe Edinburgh Postnatal Depression Scale (EPDS)-Greek version was the first tool employed [56,57,58]. The EPDS is a widely used screening scale for antepartum and postpartum depression, designed by Cox et al. [56]. It is a 10-item tool, scored on a 4-point Likert scale (from 0—the lowest grade to 3—the highest grade). A total score ranging from 0 to 30 points was calculated by summing across items. It addressed the intensity of depressive symptoms within the previous 7 days. Statistical analysis suggested a cut-off point of 11/12, with a sensitivity of 90% and a specificity of 97.2% [57]. This screening tool has been translated and validated for the Greek population by two separate research groups [57,58] and has shown a very high overall internal consistency (Cronbach’s alpha for the total scale was equal to 0.90 in the validation study of Leonardou et al. [57] and 0.80 in the validation study of Vivilaki et al. [58]). In this study the average alpha coefficient was 0.85. 

The Patient Health Questionnaire-9 (PHQ-9) [59] was the second tool used. It is a 9-item multipurpose instrument for screening, monitoring, and measuring the severity of depression symptoms over the previous 14 days (depressed mood; loss of interest or pleasure in doing things; problems with sleep, tiredness, or lack of energy; changes in appetite or weight; feelings of guilt or uselessness; problems of concentration; feelings of being slow or restless; and suicidal thoughts). Each item is rated on a 4-point scale (0 = not at all, 1 = several days, 2 = more than half the days, and 3 = nearly every day), with a total score range of 0–27. A cutoff of ≥10 was used to define possible depression. This screening tool has not been validated for the Greek population; it has only been translated for the needs of the Day Center’s participants. In this study, the average Cronbach alpha coefficient was 0.84. 

### 2.4. Analysis

The data were analyzed using SPSS version 22.0. The description of qualitative variables was provided through absolute (*n*) and relative *(%)* frequencies, while *mean* values and the *standard deviation* were used for the description of quantitative variables. The independent variables included several factors related to the psycho-emotional state of the women, their well-being characteristics from the period of pregnancy until the end of the 1st year postpartum, and data concerning their mental health state in the past. Exclusive breastfeeding, any breastfeeding duration, and the duration of breastfeeding without giving any formula were set as dependent variables. Logistic regression analyses, with a binary dependent variable (exclusive or non-exclusive breastfeeding), were performed in order to identify maternal factors associated with exclusive breastfeeding at the end of the 6th month postpartum. Moreover, a series of multiple analysis of variance (MANOVA) tests were performed to examine the relationship between socio-demographic and psycho-emotional factors and any breastfeeding duration, and between mental health factors and the duration of breastfeeding without giving any formula. 

The following clarifications should be made: (a) Exclusive breastfeeding was defined as the percentage of infants who received only breast milk and no other form of foods or liquids, except for oral rehydration solutions, drops, and syrups (vitamins, minerals, and medicines), according to WHO [60]; (b) any breastfeeding was defined as the percentage of infants who received breast milk with or without any other type of food or drink, including breast milk substitutes (non-human milk and formula) [60]; (c) the indicator “Breastfeeding without giving any formula”, corresponding to the indicator “Breastfeeding without breast-milk substitutes” that was used in recent Pan-Hellenic breastfeeding research and was considered relevant for assessing feeding practices in Greece, was also included [61]. Therefore, in this study, “Breastfeeding without giving any formula” was defined as “the percentage of infants who received breast milk with or without any other type of food or drink, except for breast-milk substitutes (non-human milk and formula)” [61]; (d) the variable “risk factors in mental health history” encompassed factors such as a family history of mental health disorders, the experience of pathological mental health symptoms in the past, traumatic life events, relationship problems with the partner (e.g., domestic violence), and poor socio-economic situation/unemployment; and (e) no objective and specific definition was given for traumatic life events. Participants had to answer the question “Has anything traumatic event happened in your life in the last year?” with a “Yes” or “No”, depending on whether they themselves thought they had experienced a traumatic event in their lives. A series of multiple analysis of variance (MANOVA) tests were performed to examine the relationship between psycho-emotional factors and exclusive or any breastfeeding duration.

## 3. Results

### 3.1. Study Characteristics

Table 1 shows the socio-demographic characteristics of the sample. The mean age of the women was 34.12 (± 3.48) years, with the majority having completed undergraduate studies (64.9%) and being married (84%). The monthly income was 501–1000 euros for approximately half of the participants (47.9%). Regarding the psycho-emotional characteristics in the women’s medical history (Table 1), about half of the women had an aggravated family mental health history (48.6%), had a risk factor in their psychiatric history (50.1%), and had sought help from a mental health professional in the past (41.5%). Only a few had had suicidal ideation (11.4%) and had suffered emotional (16.1%) and physical (9%) violence. Regarding their mental health characteristics in the current perinatal period (Table 2), approximately half of them were mentally healthy (41.7%). Additionally, one fourth of the sample had undergone psychotherapy, while a few had received medication. Several women reported having experienced a traumatic event in their lives (26.7%) and having had problems in their marriage (24.4%).

Concerning the duration of breastfeeding in our sample, it is worth mentioning that the vast majority of women initiated breastfeeding (96.3%), either exclusively (70.7%) or any breastfeeding (25.6%), at the 1st day postpartum. At the end of the 1st month postpartum, the percentage of exclusively breastfeeding women had slightly increased (74.7%), while the corresponding percentage was 44.3% at the end of the 6th month postpartum. Almost 70% of women continued any breastfeeding after the end of the 6th month and up to 24 months postpartum. Furthermore, about half of all participating women (56.1%) continued to breastfeed, without having given any formula beyond the end of the 6th up to the 24th month postpartum. The above data are not presented in table form.

### 3.2. Logistic Regression Analysis Model with Exclusive Breastfeeding at the End of the 6th Month Postpartum as the Dependent Variable

**Figure 1:**
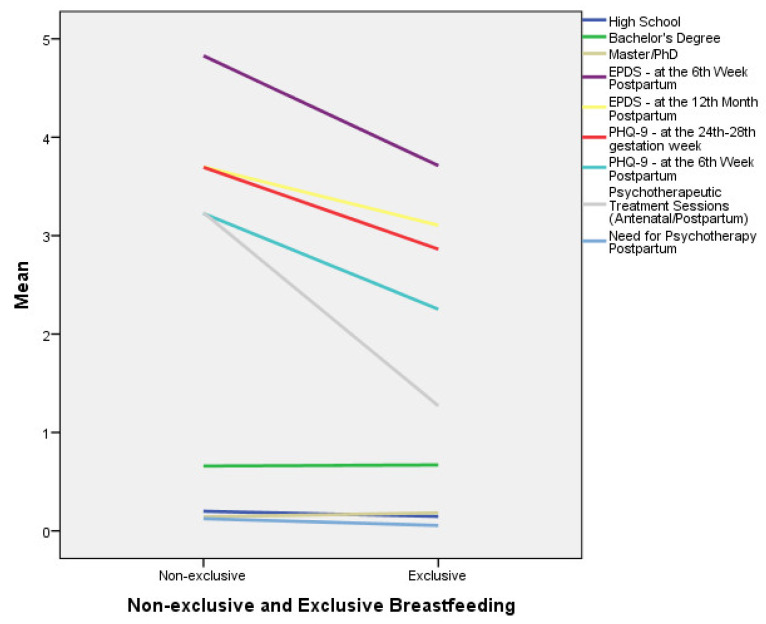
The relationship between exclusive breastfeeding at the end of the 6th month postpartum and independent variables according to the logistic regression analysis model.

Table 3 presents the results of a logistic regression analysis model that aimed to identify factors associated with exclusive breastfeeding at the end of the 6th month postpartum. Only statistically significant relationships are reported. According to this analysis, women with a lower educational level (high school graduates) have, approximately, a 29% lower chance for exclusive breastfeeding at the end of the 6th month postpartum, in comparison to those with a higher education (*p* = 0.004). Additionally, for each additional score unit of the EPDS at the 6th week postpartum, the chance for exclusive breastfeeding at the end of the 6th month postpartum appeared to decrease by 6.7% (*p* < 0.001). The same holds for the PHQ-9 at the 24th–28th gestation week, and the PHQ-9 at the 6th week postpartum, where, for each additional score unit, the chance for exclusive breastfeeding appeared to decrease by 7.8% (*p* = 0.030) and 10.8 (*p* < 0.001), respectively. Moreover, for each additional psychotherapeutic session needed antenatally, the chance for exclusive breastfeeding at the end of the 6th month postpartum appeared to decrease by 3.5% (*p* = 0.030). Finally, the need for the initiation of psychotherapeutic sessions in the postpartum period appeared to decrease exclusive breastfeeding at the end of the 6th month postpartum by 48.7% (*p* = 0.002) (Table 3). Figure 1 shows the relationship between exclusive breastfeeding at the end of the 6th month postpartum and independent variables, according to logistic regression analysis. 

### 3.3. Multivariate Analyses of Variance (MANOVA)

Table 4 presents the results of the multivariate analyses of variance (MANOVA); only statistically significant relationships are reported. The analyses revealed a significantly longer duration of breastfeeding without giving any formula (*p* = 0.035) for those participants who had completed master’s and/or PhD studies, compared to high school graduates. The proportion of variance explained was 0.6%. Regarding women’s mental health status, it appeared that participants without pathological mental health symptoms had a significantly longer any breastfeeding duration (*p* = 0.029) compared to those with increased scores for the psychometric tools, explaining 0.7% of the variance. Moreover, those who scored higher than 8 on the EPDS scale at the 6th week postpartum had a shorter any breastfeeding duration (*p* < 0.001) than those whose score was less than 8. The proportion of the variance explained was 1.9%. 

**Figure 2:**
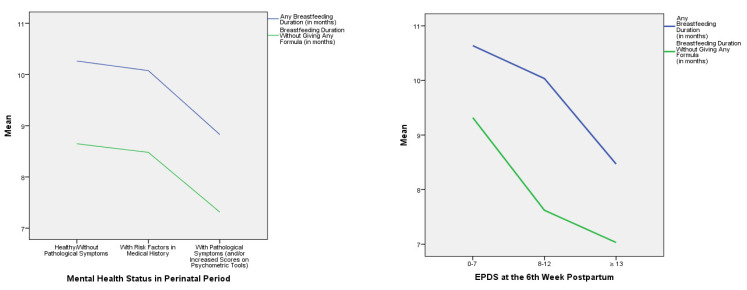

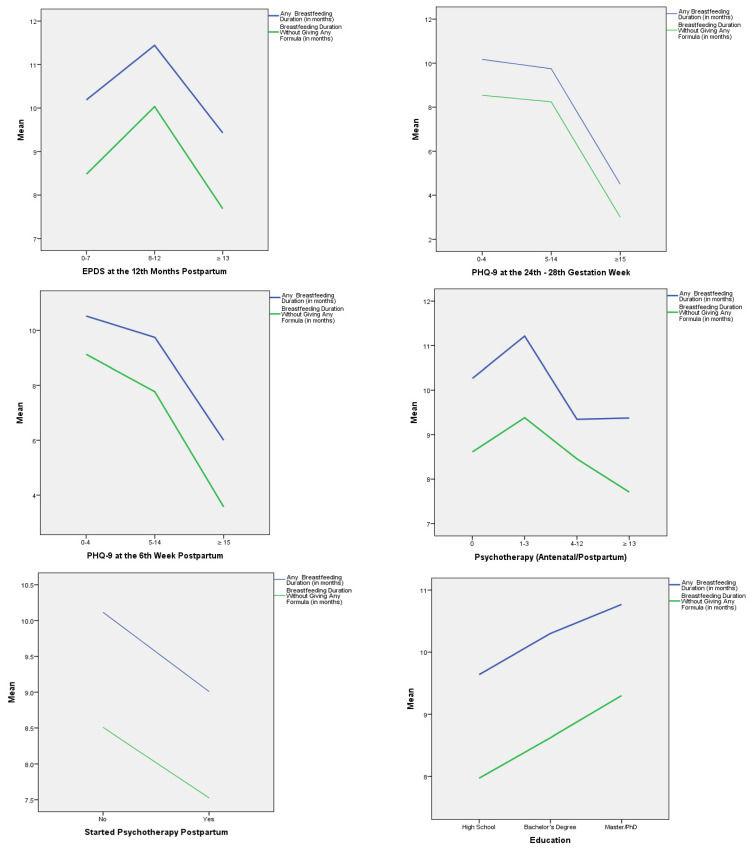
The means of any breastfeeding duration and breastfeeding duration without giving any formula according to women’s mental health characteristics in the perinatal period.

Additionally, any breastfeeding duration (*p* = 0.31) and breastfeeding duration without giving any formula (*p* = 0.20) seemed to be significantly decreased for the participants who scored higher than 8 on the EPDS scale at the 12th month postpartum, in relation to those who scored less, explaining 0.8% of the variance for any breastfeeding duration and 1% for breastfeeding duration without giving any formula. In relation to PHQ-9 scores measured between the 24th and 28th gestation week, it seems that those who scored higher than 5 had a significantly shorter any breastfeeding duration (*p* = 0.018) and breastfeeding duration without giving any formula (*p* = 0.021) than those who scored less than 5. The proportion of variance explained was 1.1% for any breastfeeding duration and 1.0% for breastfeeding duration without giving any formula. Additionally, those who scored higher than 15 on the PHQ-9 scale at the 6th week postpartum had a significantly shorter any breastfeeding duration (*p* < 0.001) and breastfeeding duration without giving any formula (*p* < 0.001) than those who scored less than 5, explaining 2% of the variance for any breastfeeding duration and 1.7% for breastfeeding duration without giving any formula. Furthermore, any breastfeeding duration (*p* = 0.020) seemed to decrease significantly for those who needed 13 or more psychotherapeutic sessions in the perinatal period, in relation to those who did not need any, with 0.4% of the variance explained. Those who started attending psychotherapy sessions postpartum showed a shorter any breastfeeding duration (*p* = 0.040). The proportion of variance explained was 0.4% (Table 4). Figure 2 shows the correlations of the means of any breastfeeding duration and breastfeeding duration without giving any formula according to women’s mental health characteristics, after multivariate analyses (MANOVA). 

## 4. Discussion

This study demonstrates a negative relationship between breastfeeding exclusivity and duration and the presence of pathological symptoms of maternal mental health from pregnancy until the 1st year postpartum. More specifically, according to the logistic regression analysis, the chance for exclusive breastfeeding at the end of the 6th month postpartum appeared to decrease (a) when the mother was of a lower educational level (*p* = 0.004); (b) with an increase of the score units for psychometric tools completed either antenatally, i.e., at the 24th–28th gestation week (PHQ-9, *p* = 0.030), or at the end of the puerperium, i.e., at the 6th week postpartum (EPDS, *p* < 0.001 and PHQ-9, *p* < 0.001); (c) with an increase in the number of psychotherapeutic sessions needed antenatally (*p* = 0.030); and (d) when the initiation of psychotherapeutic sessions in the postpartum period was necessary (*p* = 0.002). 

In addition, the multivariate analyses of variance (MANOVA) showed that a shorter any breastfeeding duration seems to be associated with (a) pathological maternal mental health symptoms during the perinatal period (*p* = 0.029), (b) increased PHQ-9 scores at the 24th–28th gestation week (*p* = 0.018), (c) increased EPDS scores in the early (at the 6th week) (*p* = 0.004) and late (at the 12th month) postpartum period (*p* = 0.031), (d) the initiation of psychotherapy postpartum (*p* = 0.040), and (e) the number of psychotherapeutic sessions needed (>13 sessions) (*p* = 0.020). Furthermore, a shorter breastfeeding duration without giving any formula seems to be associated with (a) increased PHQ-9 scores at the 24th–28th gestation week (*p* = 0.021), (b) increased PHQ-9 scores in the early postpartum period (at the 6th week) (*p* = 0.002), (c) increased EPDS scores in the late postpartum period (at the 12th month) (*p* = 0.020), and (d) the mother’s low educational level (*p* = 0.035). Therefore, this study highlights the importance of woman′s mental health during the perinatal period, considering it a key parameter for the continuation of breastfeeding (exclusive or not).

The results of this study confirm, once again, the existence of a negative relationship between the psycho-emotional pathologies of the mother in the perinatal period, and the exclusivity and continuation of breastfeeding. The aggravated mental health of women, especially when the psycho-emotional pathology persists [62], seems to contribute to the early cessation of breastfeeding, as has been described by several research studies [18,28,35,37,40,41,63,64]. Although there have been views expressed in the international literature that refer to an ambiguous association between breastfeeding and maternal mental health [37,38], and although the exact direction of this association may not have been clarified, international research constantly proves with empirical data (like that presented here) that women who are mentally burdened clearly have difficulties with breastfeeding and choose to stop it early.

In order to understand the attitude and behavior of women towards breastfeeding and to highlight the socio-demographic and medical/health factors associated with it so as to properly promote and support it, international research in recent decades has focused on specific areas, such as (a) exploring the role of existing hospital practices and highlighting the benefits of implementing the 10 steps to successful breastfeeding; (b) investigating the attitude, education, and role of healthcare professionals [65]; (c) exploring the value of prenatal education and postnatal support of women for breastfeeding [66]; (d) the adoption and implementation of new policies and legislation that are more mother-friendly and allow a mother to exercise her choice to breastfeed [65,67]; and (e) highlighting erroneous social practices and perceptions that hinder the initiation and continuation of breastfeeding [65,68,69,70]. 

Nevertheless, an issue that, to this day, does not often receive the due attention of the state, scientific communities, and healthcare professionals when assessing the reasons why women do not start or continue breastfeeding is the question of the woman’s psycho-emotional state during the perinatal period. While healthcare professionals tend to evaluate and check the breastfeeding practices applied by mothers, e.g., breastfeeding positions, time and frequency of meals, frequency of daily urination and defecation by the neonate, infant’s physical development, etc., they do not often give appropriate attention to the mothers’ worries, feelings, and mood. Although it is known that mental health disorders can undermine breastfeeding [18,26,38,39], it is often the case that professionals who take care of women in the perinatal period do not feel that they have the ability to recognize them or fail to detect the pathological symptoms of mental health in time. Moreover, it is well-known that, to a large extent, perinatal mental health disorders are not recognized and treated properly by healthcare professionals caring for pregnant women and/or new mothers [71,72,73]. 

The importance of highlighting the negative association between the initiation, exclusivity, and duration of breastfeeding, and psycho-emotional health disorders should not be questioned or underestimated, as it will potentially contribute to two important public health goals worldwide: First, improvement of the outcome of breastfeeding and, second, the early detection and timely treatment of perinatal mental health disorders. Knowing, on the one hand, that women with mental health disorders face many problems with breastfeeding [18,37,38,39,40,41] and, on the other hand, that breastfeeding is a beneficial mechanism for maternal mental health [20,26,27,28] and its early cessation foreshadows an increase in anxiety and depressive symptoms [4,20], it is clear that more breastfeeding promotion strategies that integrate the factor of maternal mental health in the perinatal period should be developed [74]. More specifically, as has been argued in other studies [74,75,76], the early detection of mental health disorders and psychosocial factors (beginning from pregnancy), as well as the early support of women who suffer from a mental health disorder or show increased scores for psychometric tools and risk factors for a disorder, should be established, in order to indirectly prevent breastfeeding-related negative developments. In addition, healthcare professionals should be even more sensitized to providing compassionate support to women who, for whatever reason, were unable to continue breastfeeding when they originally intended to do so [76], knowing that the early cessation of breastfeeding will bring about changes in the mother’s mental health and will negatively affect the already mentally burdened mothers [29,30].

At the same time, healthcare professionals who promote breastfeeding should work more purposefully on issues that are directly related to the emotions and/or mood of the woman and additionally affect breastfeeding indirectly, such as (a) an increase in the woman’s self-confidence in breastfeeding [33,37,77]; (b) improvement in her sleep [32]; (c) and the strengthening of her parental role by enhancing contact and interaction with her baby [34], as well as of her emotional involvement in the procedures relating to her baby [40,78]. Through this tactic, healthcare professionals caring for the mother and her child will not only aim to improve the duration of breastfeeding, but also to transform the breastfeeding experience, from an experience that puts more strain on a woman′s emotions and soul to one that is enjoyable and uplifts her. Therefore, the goal should not simply be the prolongation of breastfeeding, but the transformation of the breastfeeding experience into an experience that meets the mother’s expectations, desires, and needs. This is particularly important if we consider that “breastfeeding may not be the most effective or feasible option for all mothers” [74] and also that the highest risk of developing a mental health disorder was found in women who were planning to breastfeed prenatally and ultimately did not do as well as they had planned [76].

This is one of a few cases where a study has investigated the role of such a large number of psycho-emotional and psychiatric perinatal factors in breastfeeding exclusivity and duration for such a long and continuous period of time. However, some limitations must be taken into account when considering the results of this study. One limitation is the use of self-report instruments for the screening of depressive symptoms. These psychometric tools do not diagnose depression, and the fact that a woman has a high score does not necessarily mean that she also suffers from depression. However, the psychometric tools used in this study are globally accepted and recognized, and are widely used in international research for a similar purpose, i.e., the detection of pathological mental health symptoms. Furthermore, we used the cut-off scores suggested by researchers who reported a fairly satisfactory level of sensitivity and specificity for these tools. Internationally and over time, a large volume of studies has been based on findings derived from psychometric tools like the ones used in this study. 

Another limitation is that the sample of this study is not considered strictly representative of Greek mothers. It was obtained from the specific clinical context of a primary mental health facility (Day Center) located in the region of Attica. It should be mentioned, however, that about 50% of all Greeks live in this region. Therefore, given this fact, along with the large size of the sample, we can argue that our results are valuable because the research does not concern a small group of Greek women who have particular characteristics. Moreover, this study was not exclusively conducted with a population with burdened mental health. Although the facility pertains to the field of mental health, as shown in the results, approximately half of the women (41.7%) did not exhibit pathological symptoms and did not have risk factors for developing a mental health disorder or increased scores using the psychometric tools. That is because this Day Center’s chief aims are the prevention and early detection of perinatal mental health disorders in all pregnant women and mothers and not simply the treatment and cure of these health disorders. Therefore, it serves every woman who is interested in receiving midwifery-led education antenatally and support during the perinatal and lactation period.

## 5. Conclusions

This study demonstrates that exclusive breastfeeding and breastfeeding duration exhibit a significant association with a woman’s psycho-emotional health in the perinatal period. Given that in many countries internationally [6,7], including Greece [61], exclusive breastfeeding and breastfeeding duration are far from what is recommended, it is crucial to understand which factors are associated with exclusive breastfeeding and the continuation of breastfeeding. Further research is needed to understand the role that early screening and the timely treatment of psycho-emotional health disorders in all pregnant women and new mothers plays in breastfeeding and in qualitative improvement of the breastfeeding experience.

## Figures and Tables

**Table 1:** Socio-demographic and psycho-emotional characteristics in the women’s medical history.

*Scheme*			*Range*	
Age (years; mean *SD*)	34.12	3.48	21–50	
Education (*n* (*%*))				
High School	189	17.5		
Bachelor’s Degree	701	64.9		
Master’s/PhD	187	17.3		
Total/Missing	1077/3	99.7/0.3		
Marital Status (*n* (*%*))				
Married	907	84.0		
Not Married	27	2.5		
Single-Parent Family	16	1.5		
Living with a Partner	130	12.0		
Total/Missing	1080	100.0		
Monthly Income (*n* (*%*))				
0–500 euros	237	22.0		
501–1000 euros	517	47.9		
>1000 euros	305	28.2		
Total/Missing	1059/21	98.1/1.9		
Psycho-emotional Characteristics in the Women’s Medical History				
Woman’s Mental Health Symptoms before the Current Pregnancy (*n* (*%*))				
No Symptoms	450	48.9		
Anxiety	258	21.5		
Depressive	161	12.5		
Anxiety and Depressive	210	17.0		
Total/Missing	1079/1	99.9/0.1		
Aggravated Family Mental Health History (*n* (*%*))				
No	550	50.9		
Yes	525	48.6		
Total/Missing	1075/5	99.5/0.5		
Risk factors in Psychiatric History (*n* (*%*))				
No	539	49.9		
Yes	541	50.1		
Total/Missing	1080	100.0		
Help from a Mental Health Professional (*n* (*%*))				
No	632	58.5		
Yes	448	41.5		
Total/Missing	1080	100.0		
Suicidal Ideation (*n* (*%*))				
No	954	88.3		
Yes	123	11.4		
Total/Missing	1077/3	99.7/0.3		
Emotional Violence (*n* (*%*))				
No	900	83.3		
Yes	174	16.1		
Total/Missing	1074/6	99.4/0.6		
Physical Violence (*n* (*%*))				
No	983	91.0		
Yes	97	9.0		
Total/Missing	1080	100.0		

**Table 2:** Women’s mental health characteristics in the current perinatal period.

*Scheme*			*Range*	
Women’s Mental Health Status (*n* (*%*))				
Healthy/Without Pathological Symptoms	450	41.7		
With Risk Factors in Medical History	487	45.1		
With Pathological Symptoms (and/or increased scores for Psychometric Tools)	139	12.9		
Total/Missing	1076/4	99.6/0.4		
EPDS—at the 24th–28th gestation week (score; mean *SD*)	4.93	4.59	0–28	
EPDS—at the 6th week postpartum (score; mean *SD*)	4.55	4.62	0–27	
EPDS—at the 34th–38th gestation week (score; mean *SD*)	4.33	4.04	0–22	
EPDS—at the 6th month postpartum (score; mean *SD*)	3.58	4.34	0–28	
EPDS—at the 12th month postpartum (score; mean *SD*)	3.56	4.11	0–22	
PHQ-9—at the 24th–28th gestation week (score; mean *SD*)	3.25	3.25	0–24	
PHQ-9—at the 34th–38th gestation week (score; mean *SD*)	2.73	2.72	0–15	
PHQ-9—at the 6th week postpartum (score; mean *SD*)	2.93	3.46	0–27	
PHQ-9—at the 6th month postpartum (score; mean *SD*)	2.67	3.50	0–24	
PHQ-9—at the 12th month postpartum (score; mean *SD*)	2.73	3.46	0–25	
Psychotherapeutic Treatment Sessions (Antenatal-Postpartum) (number of sessions; mean *SD*)	10.58	13.45	1–75	
0 (*n* (*%*))	802	74.3		
1–3 (*n* (*%*))	137	12.7		
4–12 (*n* (*%*))	60	5.6		
≥13 (*n* (*%*))	81	7.5		
Total/Missing	1080	100.0		
Use of Psychotropic Medicine Prenatally (*n* (*%*))				
No	1073	99.4		
Yes	6	0.6		
Total/Missing	1079/1	99.9/0.1		
Started Psychotherapy Postpartum (*n* (*%*))				
No	960	88.9		
Yes	118	10.9		
Total/Missing	1078/2	99.8/0.2		
Started Taking Psychotropic Medicine Postpartum (*n* (*%*))				
No	1066	98.7		
Yes	14	1.3		
Total/Missing	1080	100.0		
Traumatic Life Events up to 12 Months Postpartum (*n* (*%*))				
No	792	73.3		
Yes	288	26.7		
Total/Missing	1080	100.0		
Marriage Problems (*n* (*%*))				
No	812	75.2		
Yes	263	24.4		
Total/Missing	1075/5	99.5/0.5		

**Table 3:** Logistic regression analysis model with exclusive breastfeeding at the end of the 6th month postpartum as the dependent variable.

	*B*	*S.E.*	*p*	*Exp(B)*
High School	−0.038	0.168	0.044	0.713
Bachelor’s Degree	0.159	0.131	0.225	1.172
Master’s/PhD	0.090	0.163	0.583	1.094
EPDS—at the 6th Week Postpartum	−0.700	0.020	*p* < 0.001	0.933
EPDS—at the 12th Month Postpartum	−0.006	0.018	0.746	0.994
PHQ-9—at the 24th–28th gestation week	−0.081	0.038	0.030	0.922
PHQ-9—at the 6th Week Postpartum	−0.114	0.030	*p* < 0.001	0.892
Psychotherapeutic Treatment Sessions (Antenatal-Postpartum)	−0.017	0.011	0.030	0.965
Started Psychotherapy Postpartum	−0.667	0.215	0.002	0.513

*Notes*: Assessment of interpretive power = 0.109; *B* = logistic coefficient; *S.E.* = standard error of estimate; *p* = significance; and *Exp(B)* = exponentiated coefficient.

**Table 4:** Multivariate analyses of variance of any breastfeeding duration and duration of breastfeeding without giving any formula according to maternal mental health characteristics in the perinatal period.

	Any Breastfeeding Duration (in months)		Breastfeeding Duration without Giving Any Formula (in months)					
	*M*	*SD*	*M*	*SD*	*F*	*df*	*p*	*η^2^*
Education *								
High School			7.58a	6.57	3.35	2	0.035	0.006
Bachelor’s Degree			8.42ab	5.46				
Master’s/PhD			9.09a	5.08				
Women’s Mental Health Status **								
Healthy/Without Pathological Symptoms	10.26a	5.41			3.56	2	0.029	0.007
With Risks Factors in Medical History	10.08ab	5.37						
With Pathological Symptoms (and/or increased Scores on Psychometric Tools)	8.83a	6.36						
EPDS—at the 6th Week Postpartum **					5.28	2	0.004	0.019
0–7	9.17a	5.49						
8–12	7.45b	5.27						
≥13	7.03b	6.54						
EPDS—at the 12th Month Postpartum								
0–7	9.98a	5.03			3.23	2	0.031	0.008
8–12	9.90b	5.30						
≥13	8.92b	5.59						
0–7			8.31a	5.33	4.00	2	0.020	0.010
8–12			8.27b	5.52				
≥13			7.27b	5.92				
PHQ-9—at the 24th–28th gestation week								
0–4	10.17a	5.49			5.63	2	0.018	0.011
5–14	9.75b	5.46						
≥15	4.50b	5.58						
0–4			8.54a	5.59	5.03	2	0.021	0.010
5–14			8.24b	5.52				
≥15			3.00b	4.67				
PHQ-9—at the 6th Week Postpartum *								
0–4			8.95a	5.52				
5–14			7.68ab	5.58	5.06	2	0.002	0.018
≥15			3.57b	4.20				
Psychotherapeutic Treatment Sessions (Antenatal-Postpartum) **								
0	10.03a	5.33			3.62	3	0.020	0.010
1–3	10.96ab	6.16						
4–12	9.18ab	5.47						
≥13	8.54b	6.11						
Started Psychotherapy Postpartum **								
No	10.11	5.48						
Yes	9.01	5.91			4.07	1	0.040	0.004

*Note*: *, statistically insignificant for any breastfeeding duration. **, statistically insignificant for breastfeeding duration without giving any formula. *Means* that share a common index (a/b) do not differ significantly from each other according to the Scheffé post-hoc criterion.

## Data Availability

Data are contained within the article.
